# Honeycomb‐like structures in sarcoidosis pathologically showing granulomas in walls of clustered bronchioles

**DOI:** 10.1002/rcr2.782

**Published:** 2021-05-19

**Authors:** Michiru Sawahata, Tamiko Takemura, Takeshi Kawanobe, Koichi Hagiwara, Chiyoko Kono, Tetsuo Yamaguchi

**Affiliations:** ^1^ Division of Pulmonary Medicine, Department of Medicine Jichi Medical University Shimotsuke Japan; ^2^ Department of Respiratory Medicine National Hospital Organization Utsunomiya National Hospital Utsunomiya Japan; ^3^ Department of Pathology Kanagawa Cardiovascular and Respiratory Center Yokohama Japan; ^4^ Department of Respiratory Medicine JR Tokyo General Hospital Shibuya Japan; ^5^ Department of Respiratory Medicine Shinjuku Tsurukame Clinic Shibuya Japan

**Keywords:** Fibrosis, honeycomb lung, non‐caseating epithelioid granuloma, sarcoidosis, traction bronchiectasis

## Abstract

In the clinical setting, it is often difficult to judge whether honeycomb‐like structures represent progression of fibrosis in pulmonary sarcoidosis or a complication by interstitial pneumonitis. This report described a valuable case in which pathology of video‐assisted thoracoscopic surgery specimens collected from the lungs with honeycomb‐like structures that were continuous with the dilated bronchioles on chest computed tomography (CT) showed granulomas in the membranous bronchiole walls, thereby demonstrating that the honeycomb‐like structures were lung lesions of sarcoidosis. Pathological features of these structures on chest CT included cystic changes attributable to incorporation of peripheral alveoli into dilated bronchioles in lobules: these findings in lung sarcoidosis were different from those corresponding to honeycomb lung in idiopathic pulmonary fibrosis/usual interstitial pneumonia. Radiological and pathological findings showed the possibility that progressive clustering of dilated bronchi and bronchioles causes cystic changes, resulting in the formation of honeycomb‐like structures as fibrosis progresses in sarcoidosis with lung involvement.

## Graphical abstract

We report a valuable case in which the pathology of video‐assisted thoracoscopic surgery specimens collected from the lungs with honeycomb‐like structures that were continuous with dilated bronchioles on chest computed tomography (CT) scans revealed granulomas in membranous bronchiole walls, demonstrating that the honeycomb‐like structures were lung lesions of sarcoidosis.

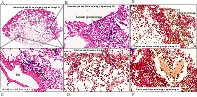

## Introduction

When honeycomb‐like structures are detected on chest computed tomography (CT) in patients with sarcoidosis with lung involvement, it is often difficult in the clinical setting to judge whether these findings represent the progression of fibrosis of lung lesions or a complication by interstitial pneumonitis such as usual interstitial pneumonia (UIP) [1]. We previously reported a case whose definite diagnosis of sarcoidosis was made based on the presence of granulomas in transbronchial lung biopsy (TBLB) specimens, and provided the first description of how honeycomb‐like structures may evolve from an initial histologically confirmed sarcoid lesion [2]. However, the number of reports on sarcoidosis with honeycomb‐like structures whose lung specimens themselves pathologically showed granulomas is limited (Table 1) [3, 4]. Moreover, pathological features of honeycomb‐like structures in sarcoidosis with lung involvement have not yet been revealed.

**Table 1:** Cases of sarcoidosis with honeycomb‐like structures on chest CT whose specimens pathologically showed granulomas.

Reported case	Sex	At detection of pulmonary granuloma		Pathological findings		Chest CT findings
		Age	Lung biopsy	Site of granuloma detection	Details of honeycomb‐like structures	Details of honeycomb‐like structures
Present case	F	63	VATS	Membranous bronchiole walls	Cyst formation by incorporation of peripheral alveoli in the dilated bronchioles	Lower lobe continuous with dilated bronchioles
Shigematsu et al. [3]	M	45	Autopsy	Connective tissue around a small bronchial wall	Dense interstitial fibrosis and honeycombing at the level of respiratory bronchioles predominantly beneath the visceral pleura	N/A
Xu et al. [4]	F	59	Lung explant	Central/peripheral peribronchiolar	Extensive, central > peripheral cysts with fibrosis. Small cysts in areas of non‐specific interstitial pneumonia. Areas of organizing pneumonia. Fibroblast foci (+)	Upper lobe with central airway dilation

CT, computed tomography; N/A, not applicable; VATS, video‐assisted thoracoscopic surgery.

Here, we report a valuable case in which the pathology of video‐assisted thoracoscopic surgery (VATS) specimens collected from the lungs with honeycomb‐like structures that were continuous with dilated bronchioles on chest CT scans revealed granulomas in membranous bronchiole walls, demonstrating that the honeycomb‐like structures were lung lesions of sarcoidosis.

## Case Report

A 54‐year‐old woman with bilateral hilar‐mediastinal lymphadenopathy and diffuse granular shadows visited the respiratory department of a regional hospital in August 2005. She suffered from chest and back pain and weight loss of 7 kg. She had a 15 pack‐year smoking history, and her past medical history included ulcerative colitis. Laboratory findings showed angiotensin‐converting enzyme 49.0 IU/L (normal level ≤ 23.7) and serum calcium 10.3 mg/dL (normal level ≤ 10.1). ^67^Ga scintigraphy showed ^67^Ga uptake in the hilar lymph nodes and lung fields bilaterally, as well as in lacrimal glands. Four months later, blurred vision developed in the left eye and uveitis was detected.

**Figure 1:**
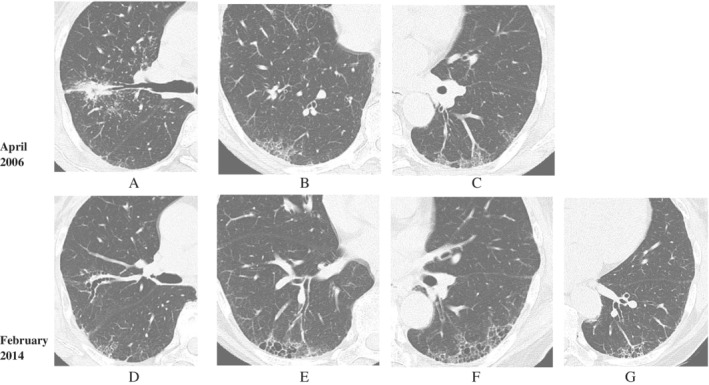
Computed tomography (CT) images taken in April 2006 (A–C). CT scans in April 2006 showed infiltrative shadows in both upper lobes and granular opacities distributed throughout the entire lung field (A), and ground‐glass or reticular pattern opacities in a wedge shape at bronchial bundle terminals on the posterior aspect of both lower lobes (B, C). CT images taken in February 2014 (D–G). CT scans in February 2014 showed the disappearance of infiltrative shadows and the appearance of bronchial dilation in both upper lobes (D). Ground‐glass opacities disappeared, and honeycomb‐like structures continuous with dilated bronchioles of which walls were thickened and appeared in both lower lobes (E–G).

In April 2006, CT scans showed various abnormalities in both upper lobes (Fig. 1A), including granular opacities distributed throughout the entire lung field, and ground‐glass opacity or reticulation in a wedge shape at bronchial bundle terminals on the posterior aspect of both lower lobes (Fig. 1B, C). She was diagnosed as having sarcoidosis based on the detection of non‐caseating epithelioid granulomas at lung lesions by TBLB as well as at skin lesions on her left hand by skin biopsy.

**Figure 2:**
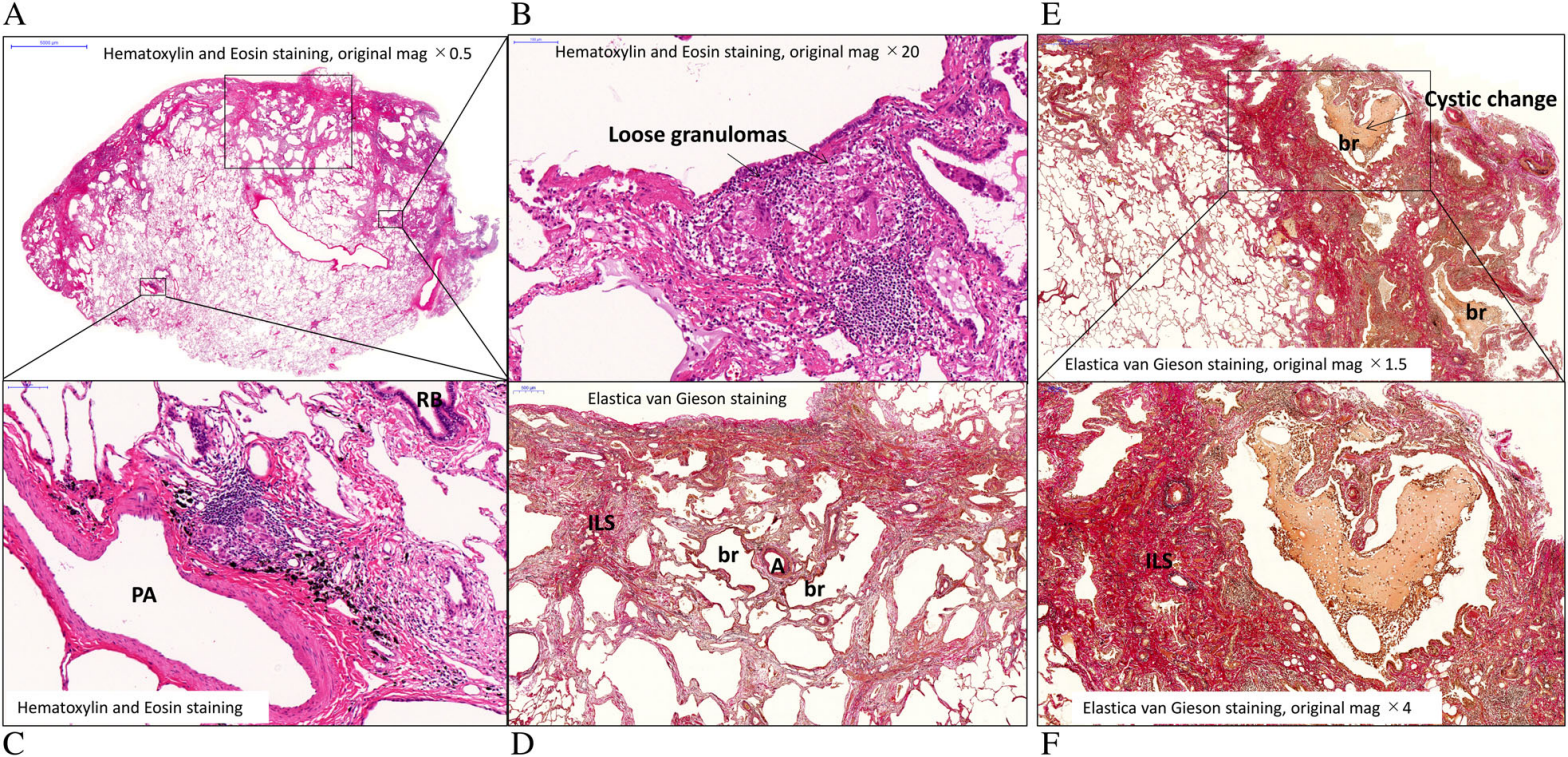
Video‐assisted thoracoscopic surgery specimens. Loosely distributed granulomas in bronchiole walls and periarterial interstitium, and fibrosis in the subpleural area and the alveolar septum (A–D). The maximum size of the cysts (dilated bronchioles) in the pathology specimen corresponding to the honeycomb lung‐like findings in the images was 2.1 mm. (A) Haematoxylin and eosin staining (low magnification). (B) Loose granulomas in bronchiole walls. (C) Loose granulomas in the periarterial interstitium with lymphocytic infiltration. (D) Subpleural fibrosis and somewhat homogeneous fibrosis of alveolar septum and bronchial dilation. Cystic lesions (E, F). (E) Aggregated cystic lesions continuous with bronchioles. (F) Cystic changes from the bronchiole level. A, artery; br, bronchiole; ILS, interlobular septum; PA, pulmonary artery.

In February 2014, CT showed the disappearance of infiltrative shadows and the appearance of bronchial dilation in both upper lobes (Fig. 1D). Also, the ground‐glass opacities disappeared, and localized honeycomb‐like structures continuous with dilated bronchioles with thickened walls appeared in both lower lobes (Fig. 1E–G). Pulmonary function test results in November 2013 were as follows: forced vital capacity (FVC) 3.61 L (%FVC 148%), forced expiratory volume in 1 sec (FEV_1_%) (G) 79.2%, and diffusing capacity of the lung for carbon monoxide (DL_CO_) 15.71 mL/min/mmHg (%DL_CO_ 93.0%). Bronchoalveolar lavage fluid results were total cell count 1.0 × 10^5^/mL, lymphocytes 19.1%, and CD4/CD8 ratio 7.0. Pathology of VATS specimens collected from the left S6 segment (Fig. 1F) confirmed loosely distributed granulomas in membranous bronchiole walls (Fig. 2A–C) and in the periarterial interstitium (Fig. 2C). Fibrosis spreading from the subpleural area towards the inside of the lobules was observed in some parts (Fig. 2D). Most cystic lesions consisted of peripheral alveoli incorporated into dilated bronchioles in the lobules (Fig. 2E, F): cyst walls did not contain many elastic fibres, and mild fibrosis and strong lymphocyte infiltration were found in some parts. Collapse induration of subpleural alveoli, which is characteristic of idiopathic pulmonary fibrosis (IPF)/UIP, was found in some parts, but the above‐mentioned pathological findings were different from those associated with honeycomb lung in IPF/UIP.

The patient was followed up without immunosuppressive treatment, and her pulmonary function values in January 2016 were found to have slightly deteriorated to FVC 3.49 L (%FVC 140.4%), FEV_1_% (G) 76.8%, and DL_CO_ 13.11 mL/min/mmHg (%DL_CO_ 78.8%). As of September 2020, she is alive with stable chest CT findings and visits a respiratory physician regularly without treatment.

## Discussion

This report described a valuable case in which pathology of VATS specimens collected from the lungs with honeycomb‐like structures on chest CT showed granulomas in the membranous bronchiole walls (Fig. 2A, B), thereby demonstrating that the honeycomb‐like structures were lung lesions of sarcoidosis.

Honeycomb‐like structures of sarcoidosis on chest CT images corresponded to pathological features including dilated bronchioles with thickened walls containing a small amount of elastic fibres and some cystic changes attributed to incorporation of peripheral alveoli into dilated bronchioles in lobules (Fig. 2E, F). These findings were different from those associated with honeycomb lung in IPF/UIP. Moreover, in the non‐cystic area, mild fibrosis progressed from the subpleural area and bronchioles (Fig. 2D) with moderate lymphocyte infiltration in some parts, and this distribution was compatible with the description of other group's previous study “fibrosis in end‐stage pulmonary sarcoid is typically distributed in a lymphangitic pattern” [1]. Indeed, the histological findings in end‐stage pulmonary sarcoidosis are reported to be distinct from those in end‐stage UIP, especially in terms of the distribution of fibrosis and the presence or absence of granulomatous inflammation [5]. Collapse induration of subpleural alveoli was found in some parts, but it was different from dense fibrosis with architectural distortion predominantly distributed in subpleural and/or paraseptal area which is a characteristic finding of IPF/UIP. Fibroblast foci were absent. According to the Official American Thoracic Society (ATS)/European Respiratory Society (ERS)/Japanese Respiratory Society (JRS)/Latin American Thoracic Society (ALAT) Clinical Practice Guideline for diagnosis of IPF [6], these findings favoured an alternative diagnosis.

In conclusion, radiological and pathological findings showed the possibility that progressive clustering of dilated bronchi and bronchioles causes cystic changes, resulting in the formation of honeycomb‐like structures as fibrosis progresses in sarcoidosis with lung involvement. However, we could not conclude that the pathological findings of this study mirror the finding of traction bronchiectasis clustering on high‐resolution CT, and further accumulation and investigation of pathological findings need to be examined in the future.

### Disclosure Statement

Appropriate written informed consent was obtained for publication of this case report and accompanying images.
